# Calcineurin-mediated dephosphorylation enhances the stability and transactivation of c-Myc

**DOI:** 10.1038/s41598-023-40412-1

**Published:** 2023-08-12

**Authors:** Takahiro Masaki, Makoto Habara, Shunsuke Hanaki, Yuki Sato, Haruki Tomiyasu, Yosei Miki, Midori Shimada

**Affiliations:** Department of Veterinary Biochemistry, Joint Faculty of Veterinary Science, Yamaguchi University, 1677-1 Yoshida, Yamaguchi, 753-8511 Japan

**Keywords:** Oncogenes, Phosphorylation, Ubiquitylation, Ubiquitylation, Cell growth

## Abstract

c-Myc, a transcription factor, induces cell proliferation and is often aberrantly or highly expressed in cancers. However, molecular mechanisms underlying this aberrantly high expression remain unclear. Here, we found that intracellular Ca^2+^ concentration regulates c-Myc oncoprotein stability. We identified that calcineurin, a Ca^2+^-dependent protein phosphatase, is a positive regulator of c-Myc expression. Calcineurin depletion suppresses c-Myc targeted gene expression and c-Myc degradation. Calcineurin directly dephosphorylates Thr^58^ and Ser^62^ in c-Myc, which inhibit binding to the ubiquitin ligase Fbxw7. Mutations within the autoinhibitory domain of calcineurin, most frequently observed in cancer, may increase phosphatase activity, increasing c-Myc transcriptional activity in turn. Notably, calcineurin inhibition with FK506 decreased c-Myc expression with enhanced Thr^58^ and Ser^62^ phosphorylation in a mouse xenograft model. Thus, calcineurin can stabilize c-Myc, promoting tumor progression. Therefore, we propose that Ca^2+^ signaling dysfunction affects cancer-cell proliferation via increased c-Myc stability and that calcineurin inhibition could be a new therapeutic target of c-Myc-overexpressing cancers.

## Introduction

c-Myc, a master transcription factor, enhances cell proliferation, differentiation, metabolism, and apoptosis^1^. c-Myc forms a dimer with Max and binds to the E-box motif to activate target-gene transcription. c-Myc expression primarily depends on mitogens and rapidly increase as cells enter the G_1_ phase of the cell cycle. Because c-Myc is sufficient to promote entry into the S phase and to overcome G1 arrest by Rb, c-Myc plays a critical role in the G1/S transition.

c-Myc expression is tightly regulated to maintain appropriate cell proliferation. The half-life of c-Myc is extremely short (approximately 30 min) and its degradation is mediated by ubiquitylation and proteasomal degradation^2,3^. Aberrant expression and activation of c-Myc are frequently observed in most human cancers. c-Myc is overexpressed in approximately 70% cancers, 20% of which show *MYC* amplification or translocation^4^.

c-Myc protein degradation is controlled by sequential phosphorylation of two critical residues: Ser^62^ and Thr^58^^5,6^. Ser^62^ phosphorylation is mediated by the Ras/MEK/ERK kinase pathway, which is a prerequisite for the subsequent phosphorylation of Thr^58^ by glycogen synthase kinase 3 β (GSK3β)^5,7^. When c-Myc is phosphorylated at these sites, its polyubiquitination is induced by F-box and WD repeat domain-containing 7 (Fbxw7) followed by subsequent proteasomal degradation^5,6,8^. Conflicting reports show that dephosphorylated Ser^62^ and phosphorylated Thr^58^ residues of c-Myc are critical for its Fbxw7-mediated ubiquitination and degradation^9^.

Thr^58^ mutations are frequently observed in Burkitt’s lymphoma cells, suggesting the involvement of Fbxw7-mediated ubiquitination and degradation pathway in cancer^10^. In leukemia and breast cancer-derived cell lines, c-Myc was found to be abnormally stabilized compared to that in controls, with higher Ser^62^ phosphorylation levels and lower Thr^58^ phosphorylation levels^11,12^. Thus, dysregulation of c-Myc degradation tuning pathways is a critical mechanism by which c-Myc is activated as an oncoprotein in human cancers.

Protein phosphatases control c-Myc expression and function; protein phosphatase 2A (PP2A) inhibits c-Myc expression by dephosphorylating Ser^62^, leading to its binding to Fbxw7^9^. Protein phosphatase 1 (PP1) along with protein phosphatase 1 nuclear target subunit (PNUTS) stabilizes c-Myc protein by modulating the dephosphorylation of 12 Ser/Thr residues, including Thr^58^ and Ser^62^^13^.

Intracellular calcium ions (Ca^2+^) trigger various physiological reactions and regulate biological processes, including cell proliferation, cell death, differentiation, muscle contraction, and neurotransmission^14,15^. Ca^2+^ signaling is essential for maintaining cellular and organismal homeostasis, and its dysregulation contributes to diseases such as heart disease, cancer, and psychiatric disorders. Calcineurin is a Ca^2+^-dependent serine/threonine phosphatase comprising catalytic and regulatory subunits, calcineurin A and calcineurin B, respectively, which is conserved in all eukaryotes. Intracellular Ca^2+^ levels increase during the G1 phase. Calcineurin is activated in response to changes in intracellular Ca^2+^ concentration and induces G1/S phase progression^16,17^. The nuclear factor of activated T cells (NFATc), the best-known target of calcineurin, is dephosphorylated to induce nuclear translocation and promote the transcription of the target genes; in T cells, this action leads to cytokine production and T-cell activation^18,19^. NFAT activates the transcription of *c-myc*^20–22^. Calcineurin controls cell proliferation by promoting the stability of cyclin D1 and estrogen receptor α (ERα), both of which induce cell proliferation^23,24^. Intracellular Ca^2+^ triggers various physiological reactions and fine-tunes biological processes, including cell proliferation, cell death, differentiation, muscle contraction, and neurotransmission^14,15^. Proper intracellular Ca^2+^ is tightly controlled by various calcium channels and calcium pumps in the plasma membrane, endoplasmic reticulum, and mitochondria. Indeed, several types of channels/pumps and calcium regulatory proteins are highly expressed in cancer. Abnormal Ca^2+^ signaling and altered Ca^2+^ concentrations are associated with tumor growth and resistance to anti-cancer therapy^14,15^. Thus, understanding abnormalities of calcium homeostasis in cancer and the appropriate control of intracellular Ca^2+^ levels is important for new anticancer-drug development. Herein, by performing comprehensive gene expression analysis, we found that intracellular Ca^2+^ activates c-Myc target genes and elucidated the molecular mechanism of this activation.

## Materials and methods

### Cell culture and reagents

MCF7 (HTB-22, ATCC), T47D (HTB-133, ATCC), and HEK293T (632180, Takara) cells were cultured in Dulbecco's modified Eagle's medium (D-MEM) (044-29765, WAKO) supplemented with 10% fetal bovine serum (FBS) (173,012, Sigma) and antibiotic–antimycotic solution (15240062, Thermo Fisher Scientific). HT29 (HTB-38, ATCC) cells were cultured in McCoy's 5A medium (16600-082; Gibco) containing 10% FBS and antibiotic–antimycotic solution. All cells were cultured at 37 °C under 5% CO_2_. Cells were treated with FK506 (S500313; Selleckchem), MG132 (S261917; Selleckchem), and CN585 (207003; Merck). FK506 was used at concentrations of 50, 25, or 12.5; MG132 at 10 μM; and CN585 at 30, 25, 15, or 7.5 μM. Cycloheximide (037-20991; FUJIFILM) was used at 50 μg/mL.

### Colony-formation assay

MCF7 cells were treated with different concentrations of verapamil for 2 weeks. Developed colonies were fixed with methanol and acetic acid and stained using 0.4% trypan blue (207-03252; Wako). Colonies were quantified by ColonyCountJ^25^.

### RNA-seq analysis

Publicly available RNA-seq data for DMSO and Amlodipine-treated A549 cells were obtained from Gene Expression Omnibus (GEO) under accession number GSE159522^26^. Raw count data was acquired using RaNA-seq^27^. Genes with less than 1 counts per million in at least 1 sample were removed, and processed counts were subjected to parametric gene set enrichment analysis (GSEA) using iDEP.96^28^. Processed counts were normalized using edgeR v 3.40.1^29–31^. GSEA was performed with Signal2Noise values for all detected genes in processed counts for indicated comparisons as the ranking metric using GSEA software version 4.3.2^32,33^.

RNA-seq data of calcineurin Aα–depleted MCF7 cells (accession no. DRA011729) were analyzed as previously described^24^. For CsA-treated cells, expression data (log2-transformed quantile-normalized expression intensities) were obtained from NCBI Gene Expression Omnibus (accession no. GSE140882) and were subjected to GSEA as previously described^34^.

### Construction of expression vectors and mutagenesis

A mammalian expression plasmid for N-terminal HA-tagged *PPP3CA* (HA-CaN Aα) was prepared by directional cloning of the full open reading frame of *PPP3CA* (NM_001130691.2) into a tag-fused pcDNA3. The *PPP3CA* mutant, P484S, was generated using the KOD-Plus-Mutagenesis Kit (SMK-101, 2wq Toyobo). The N-terminal FLAG-tagged human *c-myc* (NM_002467.6) expression vector (pCI-FLAG-c-Myc) was provided by K. Nakayama (Kyushu University).

### Dual luciferase reporter assay

MCF7 cells were transfected with 48 ng pNL[NlucP/MycMax-RE/Hygro] Vector (Promega) and 2 ng pGL4.51[luc2/CMV/Neo] Vector (Promega, E1320) using 150 ng Polyethylenimine Max (24765-1, Polyscience). Forty-eight hours after transfection, NanoLuc luciferase and firefly luciferase activity were measured using the Nano-Glo Dual-Luciferase Reporter Assay System (Promega, N1610) and Nivo S micro plate reader (PerkinElmer). The luminescence elicited by NanoLuc was normalized in relation to that of firefly luciferase.

### Lentivirus generation and infection

Lentivirus generation and infection were performed as described previously^35^. Doxycycline (Dox, 1 μg/mL; D9891, Sigma-Aldrich) was used to express shRNA. shRNA target sequences are described in Supplementary Table S1.

### Transient transfection

We transfected HEK293T cells with pCI-FLAG-c-Myc, pcDNA3 HA-PPP3CA WT, and pcDNA3 HA-PPP3CA mutants using the Polyethylenimine Max (24765-1, Polyscience) transfection reagent.

### Immunoblotting

Immunoblotting was performed as described previously^36^. After SDS-PAGE and immunoblotting were completed, images were captured using a ChemiDoc Imaging System (Bio-Rad). Antibodies used here are described in Supplementary Table S2. Target-protein band intensities were quantified using Image Lab software (Bio-Rad). c-Myc half-life was calculated using GraphPad Prism version 9 (GraphPad Software).

### RT-qPCR analysis

Total RNA was extracted using ISOGEN II (311-07361, Nippon Gene) as described previously^37^ and reverse-transcribed with random primers using the High-Capacity cDNA Reverse Transcription Kit (4368814, ABI). qPCR was performed using the FastStart Universal SYBR Green Master Mix (11226200, Roche) and a StepOnePlus real-time PCR system (Applied Biosystems). The expression levels were normalized to those of 18S ribosomal RNA (18S rRNA). Primer sequences are described in Supplementary Table S3.

### Phosphatase assay

Phosphatase assay was performed as previously described^38^. FLAG-c-Myc was incubated with or without recombinant human calcineurin (3160-CA, R&D Systems), calmodulin (208,670, Merck) in assay buffer (20 mM Tris, 10 mM MgCl_2_, 0.1 mM CaCl_2_, 1 μg/mL bovine serum albumin, pH 7.5) and incubated for 2 h at 37 °C.

### Mouse xenograft

Female NOD/Shi-SCID mice (CLEA Japan Inc.) were maintained under specific pathogen-free conditions and fed a sterilized standard diet (CE-2, CLEA Japan Inc.). HT29 cells (2 × 10^6^ cells/100 μL PBS per site) were subcutaneously transplanted into the left flank. FK506 (S500313, Selleck Chem) treatment was initiated when the tumor size reached 150 mm^3^. Mice intraperitoneally received 3 mg/kg/day FK506 or vehicle control (corn oil) once daily for 3 weeks. From dissected mice, tumor fragments were harvested and homogenized in radioimmunoprecipitation assay buffer (50 mM Tris–HCl at pH 7.5, 150 mM NaCl, 1 mM ethylenediaminetetraacetic acid, 10% sodium deoxycholate, 10% SDS, and 1% Triton X-100) supplemented with phosphatase inhibitors and protease inhibitors. The supernatant extracted by sonication was mixed with sample buffer and boiled for 5 min. The animal experiments were approved by the Committee for Animal Use of Yamaguchi University. All methods were carried out in accordance with relevant guidelines and regulations. All studies are performed in accordance with ARRIVE guidelines.

### Mutation plot and functional prediction

Somatic mutation frequency data in cancer were obtained from COSMIC v96 (released May 31, 2022)^39^. Distribution and frequency of missense mutations were visualized using MutationMapper (https://www.cbioportal.org/mutation_mapper).

Functional prediction and annotation of missense mutations were performed using the dbNSFP v4 (released July 27, 2021)^40^. Domain information was based on a previous report^41^.

### Statistical analysis

All data are representative of at least three independent experiments. All statistical analyses were performed using GraphPad Prism version 9. The data are presented as the mean ± standard error of the mean (SEM), and the paired or unpaired *t*-test was used to compare the two groups. Statistical significance was set at *p* < 0.05. Significance is indicated by * (*p* < 0.05), ** (*p* < 0.01), *** (*p* < 0.001), and **** (*p* < 0.0001) in the figures.

### Ethics statement

Approval of the research protocol by an Institutional Reviewer Board: This study was approved by Genetic Modification Safety Committee of Yamaguchi University.

### Animal studies

The animal experiments were approved by the Committee for Animal Use of Yamaguchi University. All methods were carried out in accordance with relevant guidelines and regulations. All studies are performed in accordance with ARRIVE guidelines.

## Results

### Ca^2+^ signaling is involved in c-Myc protein expression

**Figure 1:**
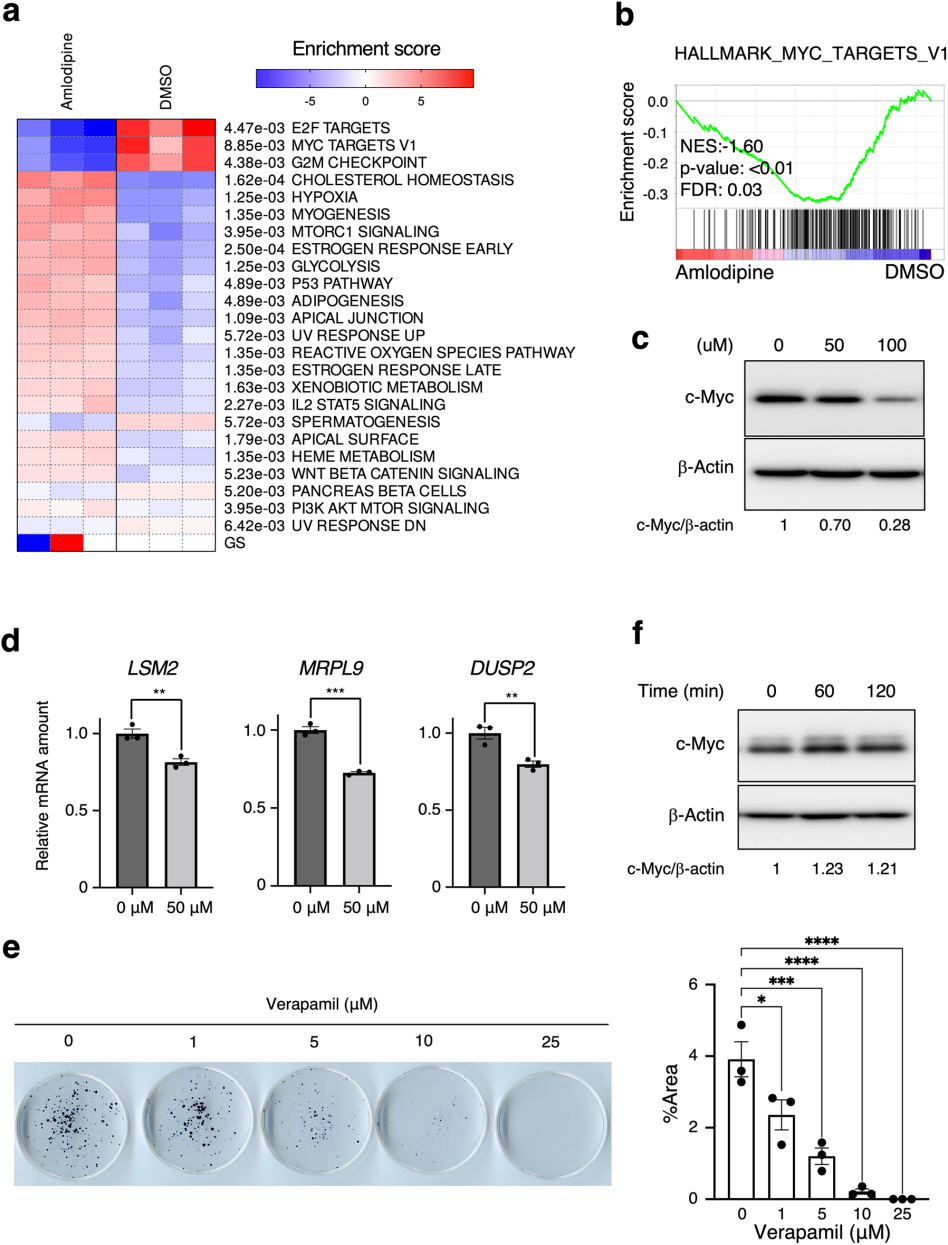
Ca^2+^ signaling is involved in c-Myc protein expression. (**a**) Significantly (*p* < 0.01) enriched biological pathways of hallmark gene sets in amlodipine–treated A549 cells compared to control (GSE159522) were revealed by parametric gene set enrichment analysis (PGSEA). (**b**) GSEA profiles of ‘’MYC TARGETS V1’’ on RNA-seq results for amlodipine treatment. (**c**)–(**d**) MCF7 cells were treated with the indicated concentrations of verapamil for 10 h. Representative immunoblot and the relative band intensity (c-Myc/β-actin) are shown (**c**). RT-qPCR analysis of c-MYC target gene expression following 10 h treatment with 50 μM verapamil (**d**). Data are the mean ± SEM from three independent experiments. **p < 0.01, ***p < 0.001 (unpaired *t*-test). (**e**) MCF7 cells were treated with indicated concentrations of verapamil for 2 weeks. The percentage of colony area to the total dish was quantified in ImageJ. *p < 0.05, ***p < 0.001, ****p < 0.0001 (Dunnett’s test). (**f**) MCF7 cells were treated with A23187 for the indicated time.

To investigate whether Ca^2+^ signaling dysfunction affects cancer-cell proliferation, we performed Gene set enrichment analysis (GSEA) on RNA-sequencing (RNA-seq) results following treatment with amlodipine, a calcium-channel inhibitor (GSE159522). c-Myc targets ("HALLMARK_MYC_TARGETS_V1" developed by on the Molecular Signatures Database) and E2F targets were greatly suppressed in amlodipine-treated cells (Fig. 1a and b), we focused on the mechanism of c-Myc target expression by intracellular Ca^2+^. To elucidate whether the c-Myc protein amount is affected by Ca^2+^, we treated MCF7 cells with Ca^2+^ channel blocker, verapamil. Verapamil inhibited c-Myc protein expression (Fig. 1c), and c-Myc target gene transcription (Fig. 1d). Consistently, verapamil inhibited colony formation (Fig. 1e). In contrast, Ca^2+^ ionophore A23187 increased c-Myc protein amount (Fig. 1f). Therefore, intracellular Ca^2+^ positively regulates the expression of the c-Myc protein and its targets.

### Calcineurin depletion attenuates c-Myc signaling

**Figure 2:**
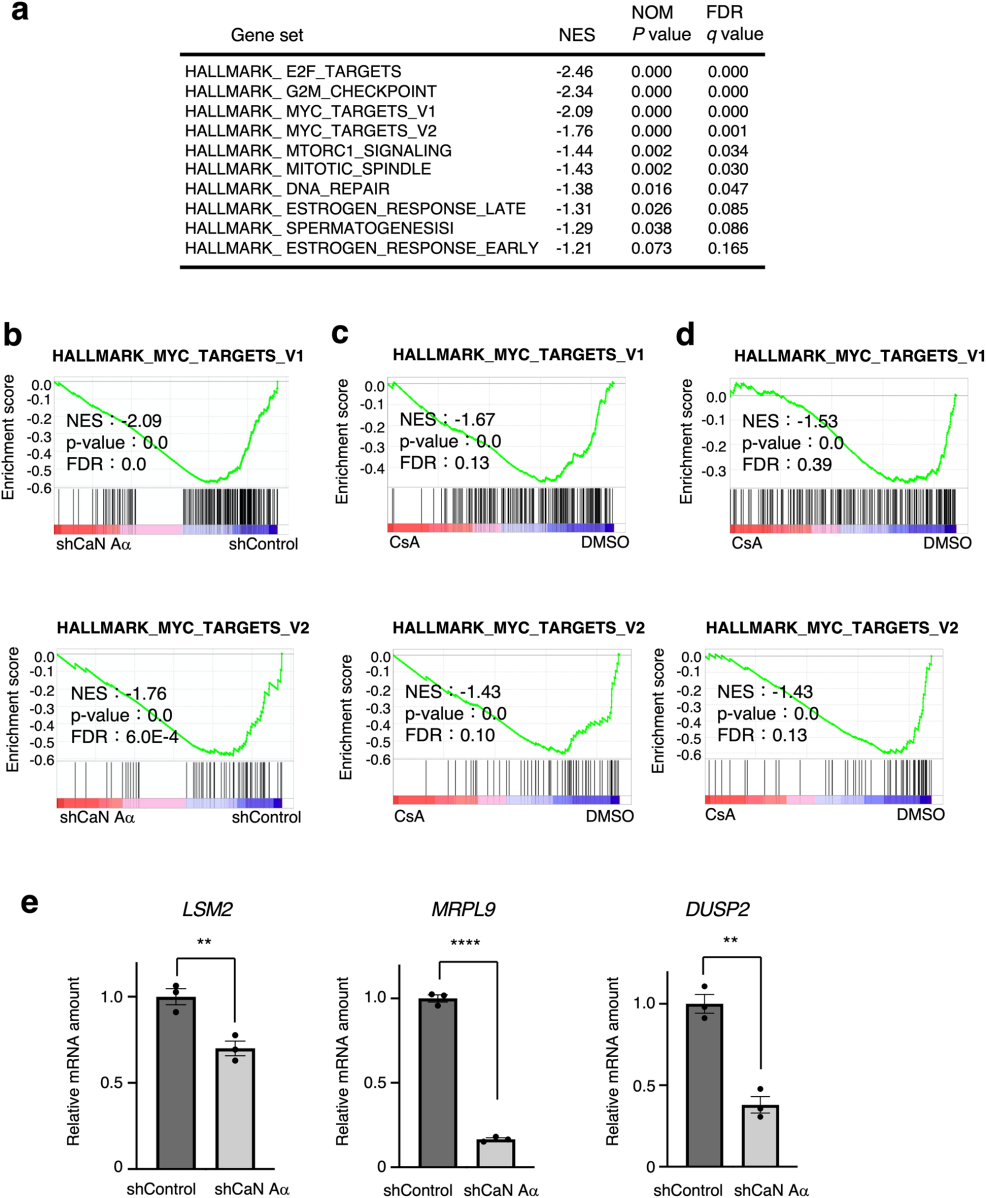
Calcineurin inhibition reduces the expression of c-MYC target genes. (**a**) Top 10 negatively enriched biological pathways from gene set enrichment analysis (GSEA) using hallmark gene sets in calcineurin Aα–depleted MCF7 cells compared with control. (**b**)–(**d**) GSEA profiles of ‘’MYC TARGETS’’ comparing MCF7 cells expressing shControl and shCaN Aα (**b**; DRA011729), HBL cells treated with CsA for 36 h (**c**: GSE140882), and HT cells treated with CsA for 36 h (**d**; GSE140882). Sizes, normalized enrichment score (NES), nominal *p* value (nP), and false discovery rate (FDR) are shown. Three biological replicates were analyzed. (**e**) RT-qPCR analysis of the expression of c-MYC target genes in the calcineurin Aα–depleted and control MCF7 cells. Data are the mean ± SEM from three independent experiments. **p < 0.01, ****p < 0.0001 (unpaired t-test).

We previously showed that calcineurin is required for cancer-cell proliferation and performed RNA-seq analysis to examine a group of genes whose expression was altered by calcineurin knockdown (DRA011729). GSEA revealed that the expression of c-Myc target genes, MYC_TARGETS_V1 and MYC_TARGETS_V2, was decreased by calcineurin depletion (Fig. 2a and b). To further investigate the effect of calcineurin on the regulation of c-Myc target genes, we analyzed the transcriptome profile of human diffuse large B cell lymphoma cells, HBL-1 and HT^42^, treated with the calcineurin inhibitor cyclosporine A (CsA) and observed that c-Myc target genes were decreased in calcineurin-inhibited cells (Fig. 2c and d). We validated these results using reverse transcriptase quantitative polymerase chain reaction (RT-qPCR) analysis and showed that c-Myc target gene expression was decreased by calcineurin depletion (Fig. 2e). Therefore, calcineurin promotes c-Myc signaling.

### Calcineurin is involved in c-Myc expression

**Figure 3:**
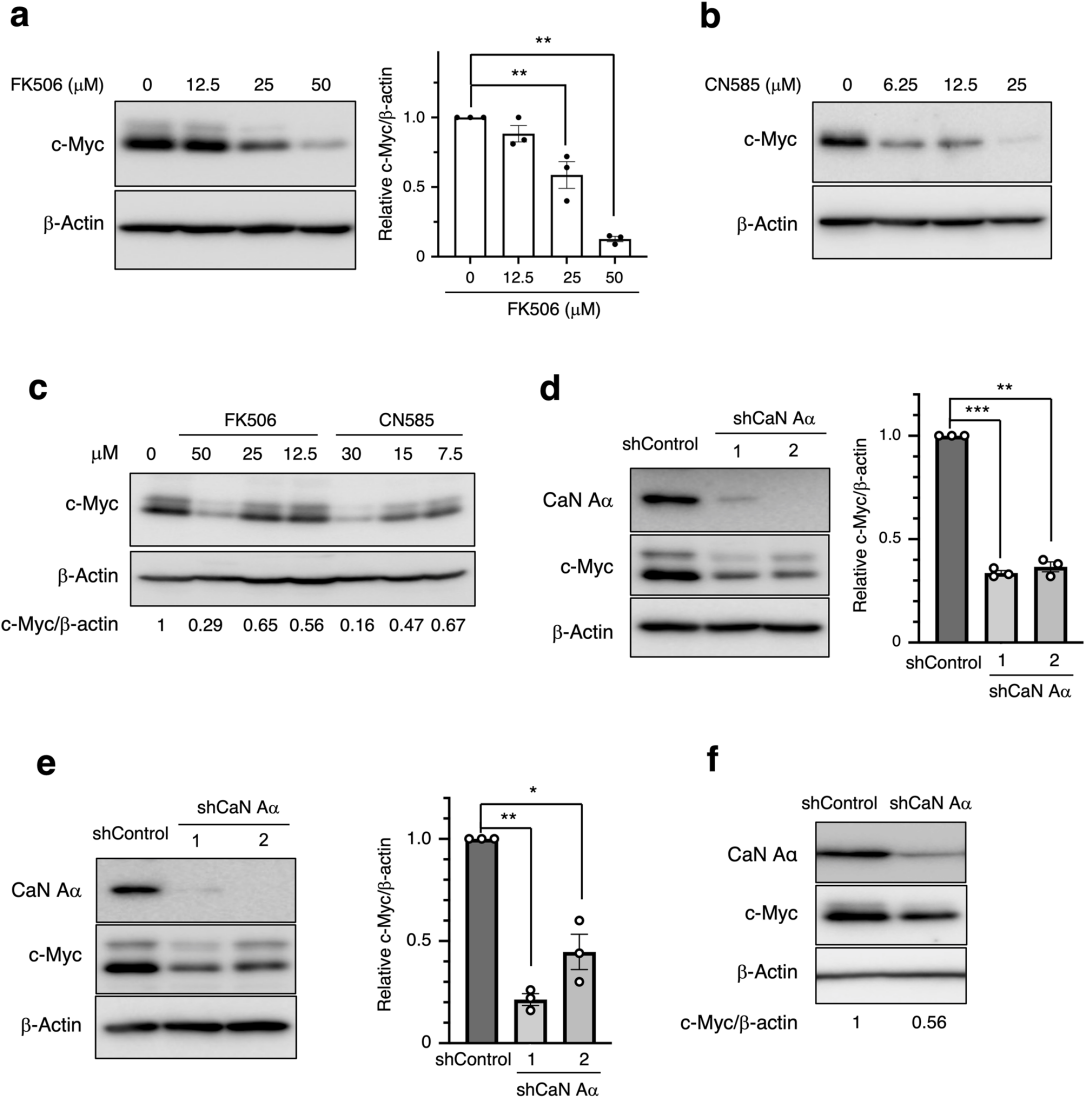
Calcineurin is involved in the c-Myc expression. (**a**) MCF7 cells were treated with the indicated concentrations of FK506 for 24 h. Total cell lysates were prepared and subjected to immunoblotting. Representative immunoblots and relative band intensity (c-Myc/β-actin) determined as the mean ± SEM from three independent experiments are shown. **p < 0.01 (Dunnett’s multiple comparisons test). (**b**) MCF7 cells were treated with the indicated concentration of CN585 for 2 h, following which total cell lysates were prepared and analyzed as in (**a**). (**c**) HT29 cells were treated with the indicated concentrations of FK506 or CN585 for 24 h and analyzed as in (**a**). (**d**)–(**e**) Lentivirus-infected MCF7 (d) or T47D (**e**) cells treated with calcineurin Aα (shCaN Aα) shRNA or luciferase (shControl) shRNAs. Two different calcineurin Aα shRNAs (1 and 2) were used. Cell lysates were then prepared and analyzed as in (**a**). The quantitative data are mean ± SEM from three independent experiments. *p < 0.05, **p < 0.01, ***p < 0.001 (paired test). (**f**) HT29 cells expressing indicated shRNAs were cultured, and total cell lysates were analyzed using immunoblotting.

Next, we aimed to confirm whether calcineurin is involved in c-Myc expression. First, we investigated effects of the calcineurin inhibitor FK506 on c-Myc expression. Immunoblot analysis revealed that c-Myc protein levels in MCF7 decreased with FK506 treatment in a concentration-dependent manner (Fig. 3a). To investigate the involvement of phosphatase activity in c-Myc expression, we treated cells with CN585, which specifically inhibits calcineurin phosphatase activity. We observed that c-Myc protein levels were decreased with CN585 treatment in MCF7 cells (Fig. 3b). Moreover, the inhibition of calcineurin by FK506 or CN585 resulted in decreased c-Myc protein levels in the human colon-cancer cell line HT29 (Fig. 3c). To test the specificity of calcineurin inhibition on c-Myc expression, two shRNAs targeting calcineurin Aα were evaluated. Calcineurin knockdown consistently reduced c-Myc protein levels in MCF7 cells (Fig. 3d), T47D cells (Fig. 3e), and HT29 cells (Fig. 3f), compared to those in controls. Therefore, calcineurin is indispensable for c-Myc expression.

### Calcineurin regulates c-Myc stability

**Figure 4:**
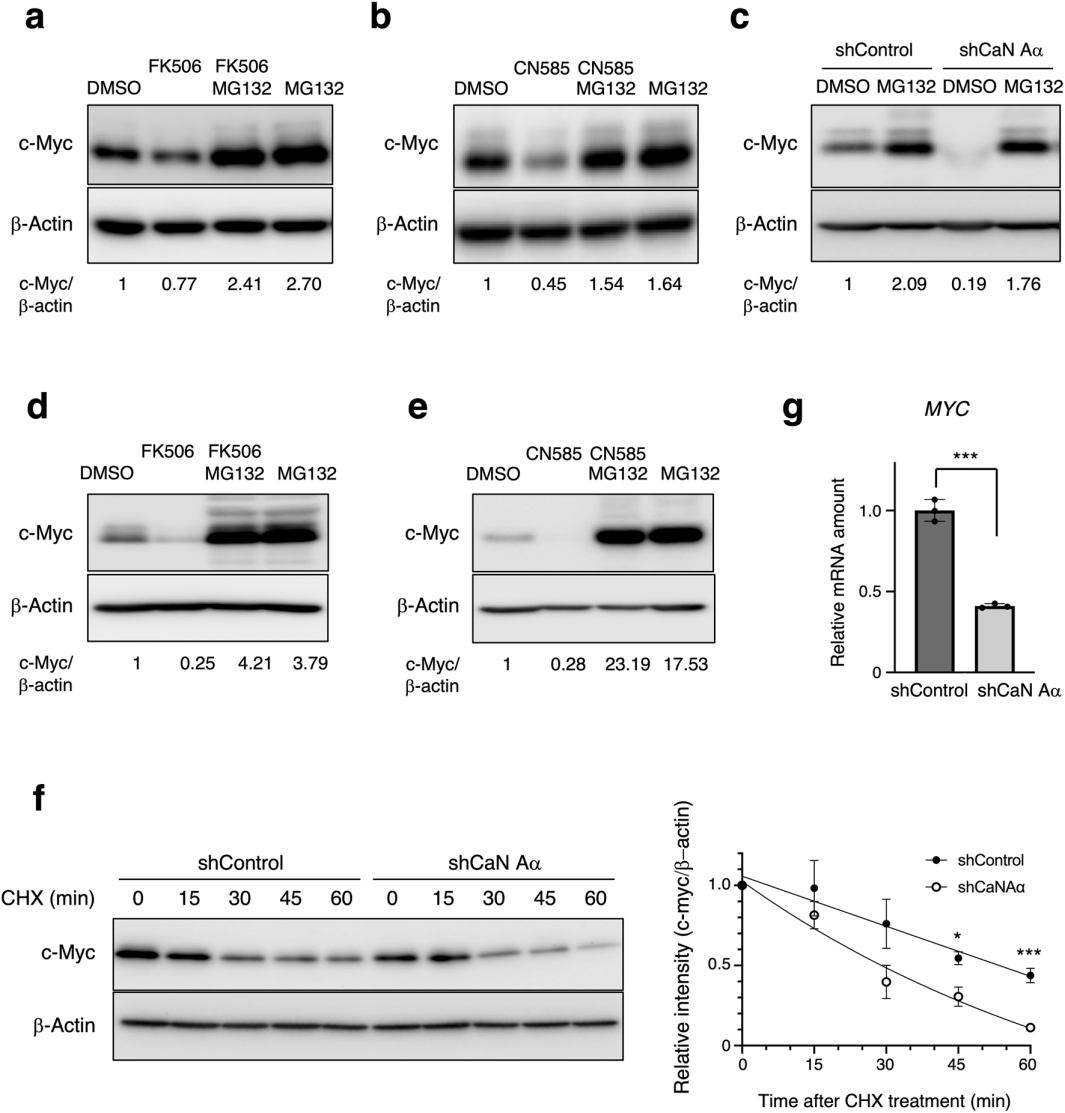
Calcineurin regulates c-Myc stability. (**a**) MCF7 cells were treated with FK506 (50 µM), MG132 (10 µM), or both for 2 h, following which total cell lysates were analyzed by immunoblotting. The relative c-Myc/β-actin band intensity ratios are shown. (**b**) MCF7 cells were treated with CN585 (30 µM), MG132 (10 µM), or both for 1 h, and total cell lysates were prepared and analyzed as in (**a**). (**c**) MCF7 cells expressing shRNAs were cultured in the presence or absence of MG132 for 24 h. Total cell lysates were analyzed by immunoblotting. (**d**)–(**e**) HT29 cells were treated with FK506 (50 µM), MG132 (10 µM), or both (**d**), or CN585 (30 µM), MG132 (10 µM), or both for 24 h, and total cell lysates were prepared and analyzed as in (**a**). (**f**) MCF7 cells expressing shRNAs were incubated with cycloheximide (CHX) for the indicated times, following which cell lysates were subjected to immunoblotting with the indicated antibodies. The relative amount of c-Myc in the blots were quantitated as the mean ± SEM values from four independent experiments. *p < 0.05, ***p < 0.001 (two-tailed Student’s t-test). (**g**) Total RNA isolated from MCF7 cells expressing indicated shRNA was subjected to RT-qPCR for analyzing *c-myc* mRNA. The RT-qPCR data are mean ± SEM from three independent experiments. ***p < 0.001 (unpaired t-test).

To determine whether the decrease in c-Myc levels induced by calcineurin inhibition was due to ubiquitin–proteasome pathway-mediated protein degradation, we evaluated the effect of the proteasome inhibitor MG132. Treatment of MCF7 cells with MG132 prevented the decrease in c-Myc protein following treatment with FK506 (Fig. 4a), CN585 (Fig. 4b), or calcineurin depletion (Fig. 4c). Similar results were obtained for HT29 cells (Fig. 4d and e). Next, we determined c-Myc stability following calcineurin knockdown using a cycloheximide (CHX) chase assay. Stability of c-Myc protein decreased in calcineurin-depleted MCF7 cells compared to control cells (Fig. 4f), suggesting that calcineurin promotes c-Myc stability by inhibiting the ubiquitin–proteasome pathway.

Since the NFATc transcription factor, which acts downstream of calcineurin, was shown to activate *c-myc* transcription^20–22^, we examined the relative amounts of *c-myc* mRNA by performing RT-qPCR. A significant reduction in *c-myc* mRNA level was observed in calcineurin-depleted cells (Fig. 4g), suggesting that decreased c-Myc protein expression following calcineurin depletion was attributable to regulation at protein and mRNA levels.

### Calcineurin increases c-Myc stability by dephosphorylating Thr^58^ and Ser^62^

**Figure 5:**
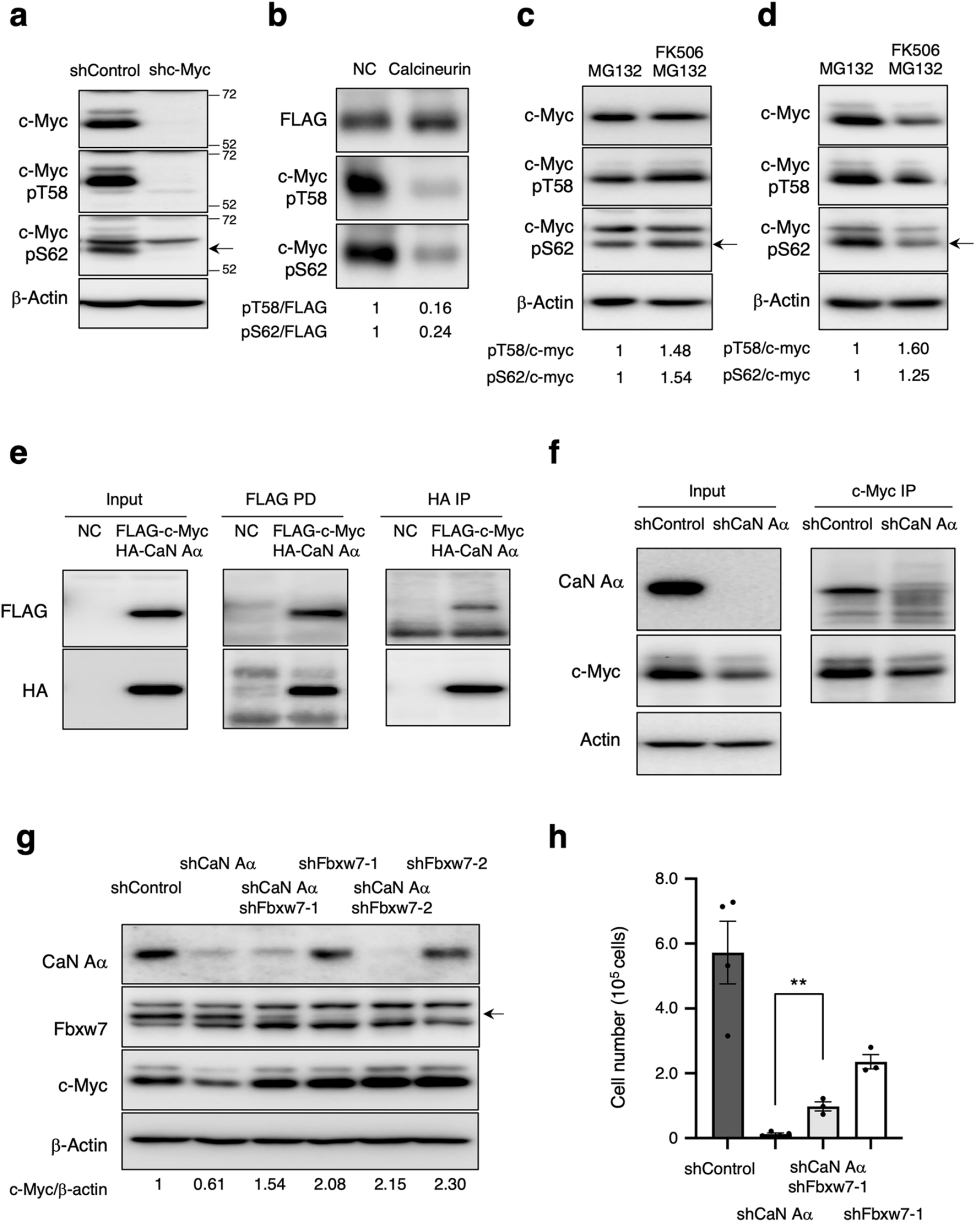
Calcineurin increases c-Myc stability via dephosphorylating Thr^58^ and Ser^62^. (**a**) MCF7 cells expressing shControl or shc-Myc were cultured, and total cell lysates were analyzed by immunoblotting; asterisks indicate non-specific bands. (**b**) Phosphatase assay performed with immunoprecipitated FLAG-tagged c-Myc with or without recombinant calcineurin and calmodulin. The relative phosphorylated c-Myc/FLAG band intensity ratio was determined. (**c**)–(**d**) MCF7 cells treated with FK506 (50 µM) with/without MG132 (10 µM) for 4 h (**c**) and HT29 cells treated with FK506 (50 µM) with/without MG132 (10 µM) for 1 h (**d**) were subjected to immunoblotting. (**e**) HEK293T cells were transiently transfected with expression vectors for HA-tagged *PPP3CA* (HA-CaN Aα) and FLAG-tagged *c-myc* (FLAG-c-Myc), or with the corresponding empty vector (NC), for two days, after which total cell lysates were prepared and subjected to immunoprecipitation (IP) with antibodies against HA or FLAG. (**f**) The MCF7 expressing shRNA cell lysates were prepared, and c-Myc was immunoprecipitated. The association of CaN with c-Myc was analyzed by immunoblotting. (**g**) MCF7 cells expressing indicated shRNAs were collected, and the total cell lysate was analyzed by immunoblotting. (**h**) MCF7 cells expressing the indicated shRNA were cultured in the presence of Dox. After four days, cell numbers were counted. Data are mean ± SEM from three independent experiments. **p < 0.01 (unpaired t-test).

Phosphorylation of Thr^58^ and Ser^62^ in c-Myc facilitates its ubiquitination by the E3 ligase Fbxw7 and subsequent proteasomal degradation^5,6^. Given that PP2A and PP1 can dephosphorylate these sites^9,13^, we next evaluated whether calcineurin also contributes to dephosphorylation. We first confirmed the specificity of commercial c-Myc phospho-antibodies using c-Myc-depleted cell lysates as controls (Fig. 5a). Next, we tested whether calcineurin mediates c-Myc dephosphorylation at Thr^58^ and Ser^62^ using an in vitro phosphatase assay. Recombinant calcineurin reduced phosphorylation at both sites, indicating that calcineurin dephosphorylated c-Myc at Thr^58^ and Ser^62^ (Fig. 5b). To examine the effect of calcineurin on c-Myc phosphorylation in vivo, we examined the phosphorylation status of Thr^58^ and Ser^62^ in calcineurin-inhibited cells treated with FK506 in the presence of MG132. We observed that calcineurin inhibition increased phosphorylation of Thr^58^ and Ser^62^ in MCF7 cells (Fig. 5c). Similar results were obtained for HT29 cells (Fig. 5d). Therefore, calcineurin plays a crucial role in dephosphorylating both pThr^58^ and pSer^62^ in vivo, consistent with the in vitro phosphatase-assay results. To investigate the potential association between c-Myc and calcineurin, we co-transfected with expression vectors encoding FLAG-tagged *c-myc* and hemagglutinin epitope (HA)-tagged *PPP3CA* into HEK293T cells and confirmed their interaction by reciprocal co-immunoprecipitation analysis (Fig. 5e). We also detected an interaction between endogenous c-Myc and calcineurin (Fig. 5f).

### Fbxw7 depletion suppresses c-Myc degradation in calcineurin knockdown cells

Several E3 ligases involved in c-Myc degradation have been reported, among which FBXW7 E3 ligase is an important regulator. To clarify whether c-Myc degradation induced by calcineurin inhibition depends on Fbxw7, we knocked down *FBXW7* using two independent shRNAs and examined its effect on c-Myc degradation following calcineurin depletion. The reduction in c-Myc by calcineurin knockdown was restored by Fbxw7 knockdown (Fig. 5g). These results indicated that c-Myc degradation following calcineurin inhibition is mediated by Fbxw7. Calcineurin inhibition represses breast cancer-cell proliferation, especially at the G1/S transition phase^23^. One reason may be the degradation of c-Myc, a master transcription factor for proliferation. Therefore, we explored the effect of c-Myc degradation by calcineurin inhibition on cell proliferation by knocking down *Fbxw7*. Consistent with a previous report, depletion of Fbxw7 led to slower growth compared to that in the control^43,44^ (Fig. 5h). Notably, knockdown of *Fbxw7* partially restored the growth arrest observed in calcineurin knockdown cells. Collectively, inhibition of calcineurin resulted in Fbxw7-dependent degradation of c-Myc.

### Mutations in the auto-inhibitory domain of calcineurin increase c-Myc protein expression

**Figure 6:**
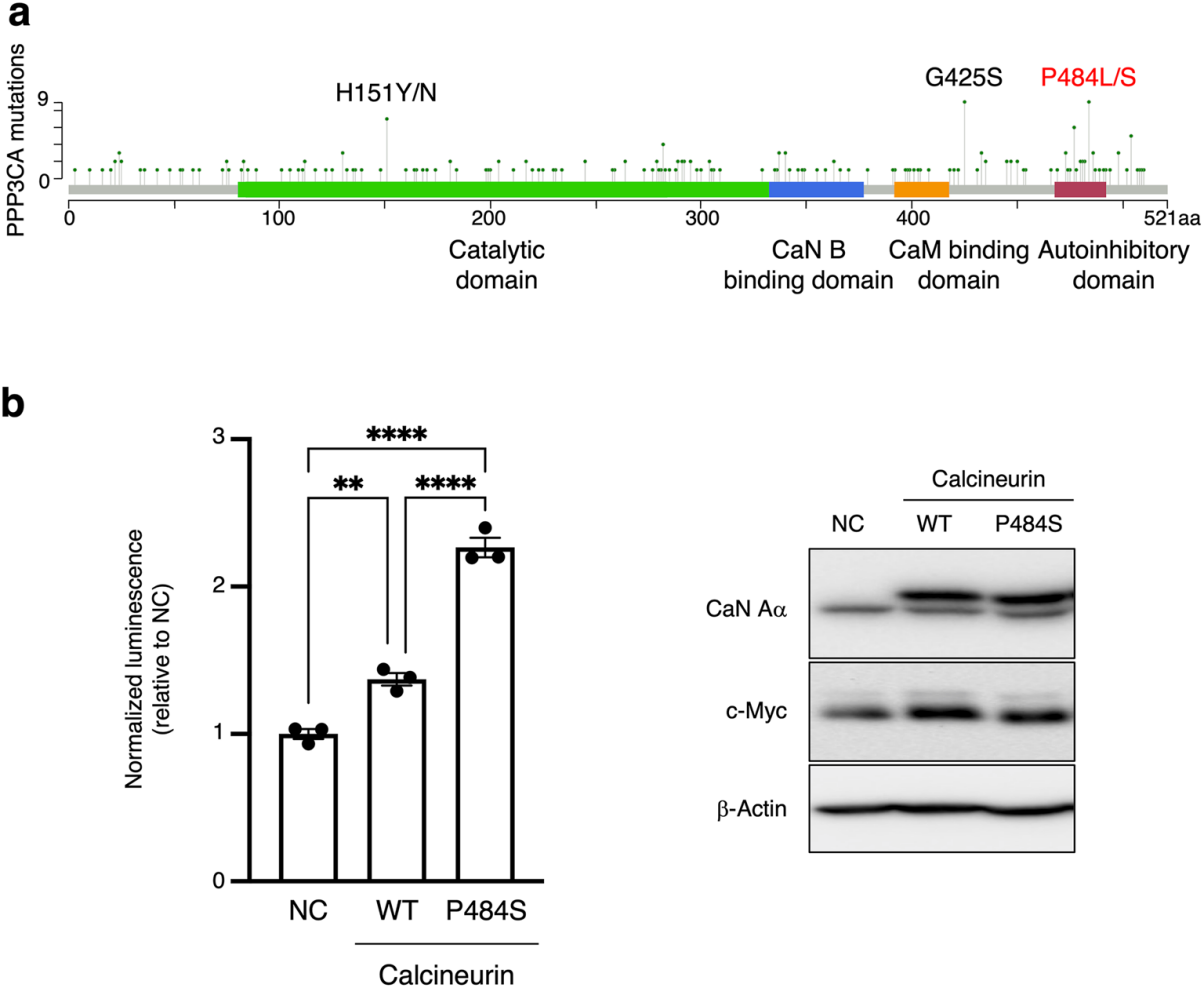
Mutations in the auto-inhibitory domain of calcineurin increase c-Myc protein expression. (**a**) Distribution and frequency of missense somatic mutations of PPP3CA in cancer were obtained from the COSMIC database and visualized by MutationMapper. (**b**) MCF7 cells stably expressing calcineurin WT or P484S were cultured for 1 d. Cells were transiently transfected with Myc-Max-RE-Nluc reporter plasmids. After 48 h, luciferase activity was measured (left). Results from three independent experiments are shown. Total cell extracts were subjected to immunoblotting (right). Data are expressed as the mean ± SEM. **P < 0.01, ****P < 0.0001 (Tukey’s test).

Calcineurin has an autoinhibitory domain at its C-terminus that negatively regulates phosphatase activity. Calcineurin hyperactivation is associated with mutations^17^. Analysis of the Catalogue of Somatic Mutations in Cancer (COSMIC) database revealed that the missense mutations in P484, located in the autoinhibitory domain, is frequent somatic mutations in cancer (Fig. 6a). Functional prediction and annotation of genomic mutations underlie P484S/L were performed using dbNSFP v4^40,45^; these mutations were found to be likely to affect calcineurin function (Supplementary Table S4). Therefore, we constructed a mutant, P484S, and examined its effect on c-Myc transcriptional activity. Wild-type (WT) calcineurin expression increased c-Myc activity, and P484S mutant expression enhanced transcriptional activity more than WT expression (Fig. 6b). Therefore, the P484 mutation may increase calcineurin phosphatase activity, thereby increasing c-Myc protein stability and transcriptional regulation activity. Collectively, we identified one critical amino acid in the auto-inhibitory region that controls c-Myc expression.

### Calcineurin inhibition suppresses c-Myc expression in vivo

**Figure 7:**
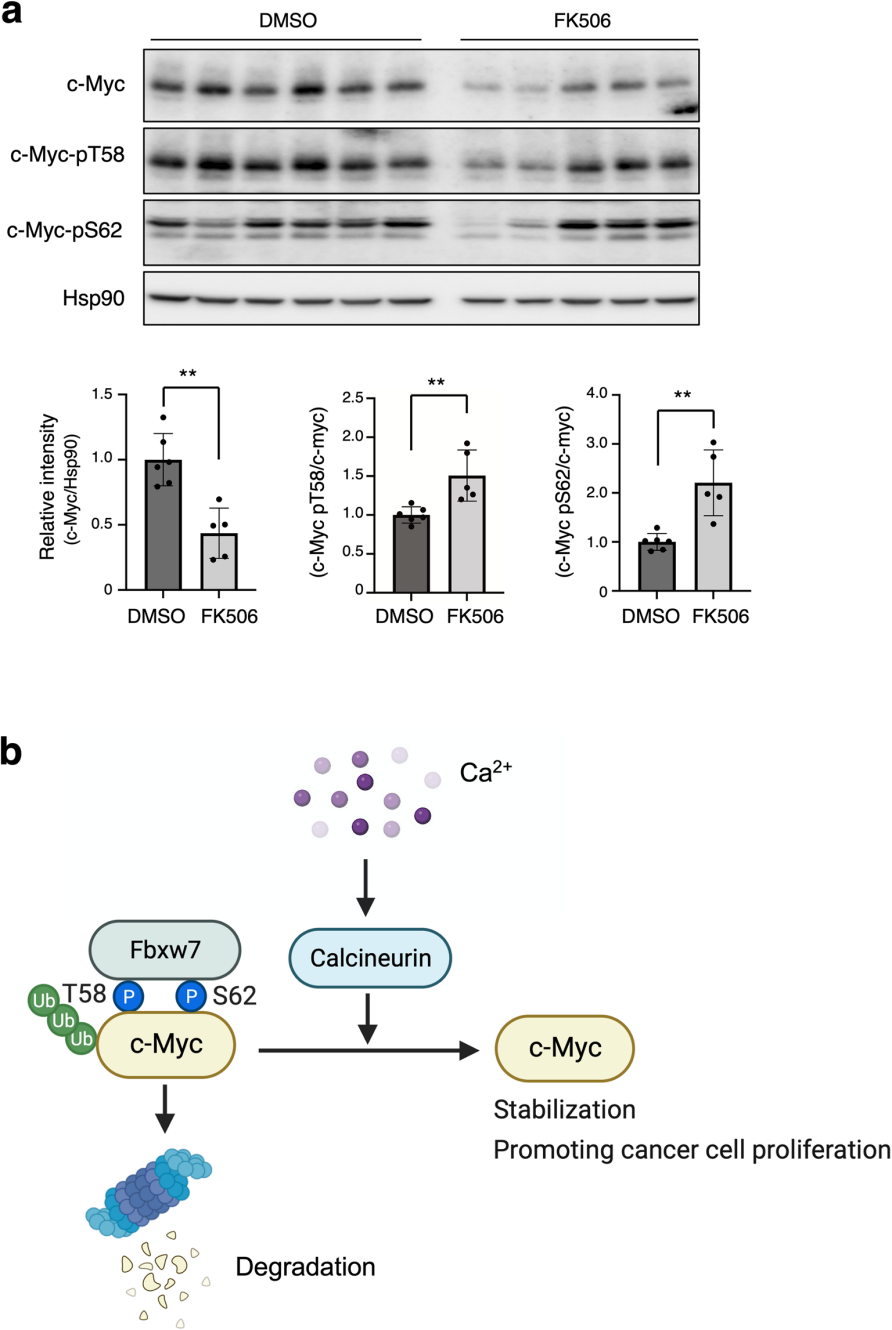
Calcineurin inhibition suppresses tumor growth in vivo. (**a**) HT29 cells were transplanted into the flank of NOD/Shi-SCID mice. Xenograft tumors post-treatment on day 21 were subjected to immunoblotting. Error bars represent mean ± SEM. **p < 0.01 (unpaired t-test). (**b**) Proposed model of c-Myc expression through dephosphorylation by calcineurin.

FK506 inhibited cancer progression in a mouse xenograft model of bladder cancer^46^. We examined the expression of c-Myc following treatment with FK506 in vivo using a mouse xenograft model. HT29 cells were subcutaneously implanted into the flanks of NOD/Shi-SCID mice. FK506 was injected daily as the tumor volume reached approximately 150 mm^3^. After 3 weeks of treatment, protein was extracted from the collected tumors, and c-Myc protein levels were examined. We observed that c-Myc protein levels decreased in FK506-treated tumors with increased phosphorylation of Thr^58^ and Ser^62^ (Fig. 7a). Therefore, calcineurin inhibition impaired c-Myc dephosphorylation and promoted c-Myc degradation.

## Discussion

In this study, by performing comprehensive gene expression analysis, we found that intracellular Ca^2+^ activates c-Myc target genes, and elucidated the molecular mechanism of this activation. Ca^2+^ signaling triggers intracellular biochemical responses by activating various calcium-binding proteins such as calmodulin, calcineurin, and CAMKII. We demonstrated that intracellular Ca^2+^ concentration modulates c-Myc stability. Essentially, calcineurin, promotes c-Myc stability via dephosphorylating c-Myc at Thr^58^ and Ser^62^ (Fig. 7b). We confirmed that calcineurin inhibition suppressed c-Myc protein levels, whereas treatment with MG132, a proteasome inhibitor, restored c-Myc protein levels, suggesting that calcineurin inhibits c-Myc degradation via ubiquitin–proteasome system. Indeed, RNA-seq analysis revealed that the expression of c-Myc targeted genes was inhibited in calcineurin-depleted cells, suggesting the involvement of calcineurin in c-Myc function. The translational modifications of c-Myc, especially the phosphorylation of two residues, Thr^58^ and Ser^62^, are particularly important for c-Myc stability. We observed that calcineurin dephosphorylates both the residues in vitro and in vivo.

Ser^62^ phosphorylation stabilizes c-Myc by preventing Fbxw7 binding; in contrast, Thr^58^ phosphorylation leads to c-Myc degradation by promoting Fbxw7 binding^7,47^. The relationship between phosphorylation and the subsequent Fbxw7-mediated degradation is complex; c-Myc binding to Fbxw7 requires Ser^62^ dephosphorylation^9^. However, another report demonstrated that Ser^62^ phosphorylation increased the affinity of Thr^58^ phosphorylated c-Myc for Fbxw7^48^, consistent with previous findings showing that phosphorylation of both sites in c-Myc is indispensable for its binding to Fbxw7^5,6^. In addition, phosphorylation of Thr^244^ and Thr^248^ and of Thr^58^ and Ser^62^, two dephosphorylated degrons, triggers binding to Fbxw7 and subsequent polyubiquitination ^48^. In a previous report, Wang et al. found that when ectopically expressed c-Myc T58A and c-Myc S62A proteins showed a higher protein level and higher half-life compared to c-Myc WT^49^. These observations are consistent with the finding that calcineurin stabilizes c-Myc by dephosphorylating Thr^58^ and Ser^62^.

We examined the relationship between these two critical phosphorylation sites and calcineurin activity. We observed that the phosphorylation of Thr^58^ and Ser^62^ increased in calcineurin-inhibited cells. Furthermore, we confirmed calcineurin binding to c-Myc and the direct dephosphorylation of both the residues in c-Myc using an in vitro phosphatase assay. Consistent with these results, we also observed decreased c-Myc expression in tumors treated with FK506, accompanied by increased phosphorylation of both the residues in mice transplanted with a colon cancer cell line. These findings are supported by the fact that FK506 and cyclosporin A induce apoptosis in leukemia cells and inhibit tumor progression.

Distinct protein phosphatases fine-tune c-Myc stability and dephosphorylation. PP2A inhibits c-Myc by dephosphorylating Ser^62^^9^. In another study on breast cancer cells, the Eya3-PP2A complex was found to stabilize c-Myc by dephosphorylating Thr^58^^50^. PP1 and its regulatory subunit, PNUTS, are involved in the dephosphorylation of at least 12 Ser/Thr residues (Thr^58^, Ser^62^, Ser^71^, Ser^81^, Ser^151^, Ser^159^, Ser^161^, Ser^314^, Thr^315^, Ser^344^, Ser^347^, and Ser^348^) of c-Myc and promote its stabilization and chromatin binding^13^. Although calcineurin and PP1 dephosphorylate Thr^58^ and Ser^62^, respectively, and PP2A dephosphorylates Ser^62^, the mechanism by which multiple phosphatases function to phosphorylate the same residues remains unclear. It has been previously reported that c-Myc transcriptional activity is induced by phosphorylation at Ser^62^. In the present study, we found that calcineurin dephosphorylates both Thr^58^ and Ser^62^ and increases the expression of c-Myc protein; however, it is unclear how c-Myc functions when Ser^62^ is dephosphorylated. In another study, Moser et al. reported that IKKα increases the transcriptional activity of c-Myc by phosphorylating Ser^67^ and Ser^71^ of c-Myc. By examining the effects of IKKα inhibition on Thr^58^ and Ser^62^, they found that IKKα inhibition did not affect Ser^62^ phosphorylation but enhanced Thr^58^ phosphorylation. Based on these results, the authors speculated that IKKα-induced phosphorylation of Ser^67^ and Ser^71^ inhibits Thr^58^ phosphorylation by altering the GSK3β recognition domain^51^. The transcriptional activity of c-Myc, therefore, could be regulated by several phosphorylation sites. For c-Myc, in which Ser^62^ is dephosphorylated by calcineurin, it is necessary to examine how the phosphorylation state of other sites involved in transcriptional activity is altered.

We found that dysfunctional Ca^2+^ signaling affects cancer cell proliferation via increased c-Myc stability mediated by calcineurin. Intracellular calcium may activate both calcineurin and ERK, the first dephosphorylating c-Myc Ser^62^ and the second phosphorylating c-Myc Ser^62^. We discuss this potentially conflicting regulation of Ser^62^ phosphorylation by increased Ca^2+^ levels. Of note, NDMA receptor regulates both activation and inactivation of ERK. Paul et al. reported that a rapid initial increase in Ca^2+^ upon NR2A-NMDAR activation in turn induces ERK via transient and rapid phosphorylation, whereas a delayed and substantial Ca^2+^ increase mediated by NR2B-NMDAR activates calcineurin. The dephosphorylation of STEP by calcineurin activates STEP, which inactivates ERK^52,53^. These findings suggest that there is a time lag between the initial Ca^2+^ influx by NR2A-NMDARs which temporarily activates ERK, and the subsequent substantial Ca^2+^ influx by NR2B-NMDARs which causes calcineurin activation and inactivates ERK by dephosphorylation of STEP. Based on these notions, it is possible that there is a time lag between the phosphorylation of c-Myc Ser^62^ by ERK and its dephosphorylation by calcineurin. It is necessary to study the time course of the change in the phosphorylation level of c-Myc Ser^62^ due to increased Ca^2+^ levels in the future.

Calcineurin is aberrantly activated in various cancer types^17^. Calcineurin hyperactivation is associated with mutations in some cancers. Calcineurin contains an autoinhibitory domain at its C-terminus, which inhibits phosphatase activity. In murine T-cell lymphoma cell lines, the Asp^477^ of this autoinhibitory domain is mutated to Asn, thereby hyperactivating calcineurin^54^. Furthermore, SML B-cell lymphoma cell lines express a truncated form of calcineurin catalytic subunit A, which results in the constitutive activation of calcineurin^55^. We observed that mutations in P484 within the autoinhibitory domain are the most prevalent in human cancer, suggesting that activated calcineurin is associated with tumorigenesis. Recently, we reported that high calcineurin expression correlates with poor prognosis after endocrine therapy of patients with ER-positive breast cancer, indicating that the selective inhibition of calcineurin may be effective in treating such cancers^23,24^. Cyclin D1 overexpression has been observed in many cancers and is correlated with malignancy^56^. Estrogen receptor α (ERα) is highly expressed in a large subset of breast cancer cases. Calcineurin stabilizes cyclin D1 and ERα by dephosphorylating Thr^286^ and Ser^296^, respectively^23,24^. Collectively, our data provide mechanistic insights into the regulation of c-Myc stability by controlling its dephosphorylation by calcineurin, and propose that calcineurin inhibition may be an effective method for cancer therapy.

Although several roles of calcineurin in cancer are discussed here, calcineurin is also an important factor in the regulation of the immune response gene expression. FK506, a calcineurin inhibitor, is used as an immunosuppressive agent in the treatment of autoimmune diseases and post-transplant rejection of organs^57^. Thus, it is possible that calcineurin inhibitors suppress cancer immunity and promote tumorigenesis. However, other studies have shown that FK506 inhibits the growth of oral squamous cell carcinoma^58^. Further studies are needed to elucidate the mechanisms by which calcineurin specifically interacts with factors that accelerate cell proliferation, such as c-Myc, CyclinD1, and ERα. Furthermore, the relationship between calcineurin upstream factors, Ca^2+^ channels, and cancer requires to be investigated.

Ca^2+^ signaling modulates various physiological responses in vivo, and Ca^2+^ channel blockers are currently used clinically for the treatment of hypertension, angina pectoris, and arrhythmias. Recently, Ca^2+^ signaling has also been shown to play an important role in cancer, suggesting that Ca^2+^ channels may be potential therapeutic targets in cancer. Cancer cells express Ca^2+^ channels that are not present in normal cells, and changes in their expression levels are frequently observed. Certain Ca^2+^ channels have increased expression levels in several cancer types, including prostate, thyroid, colon, ovarian, and breast cancer, and their inhibition has been reported to suppress tumorigenesis^59^. However, blockade of Ca^2+^ channels may cause side effects through the inhibition of cardiac stimulation, conduction, and vasodilation. Therefore, further understanding, including elucidation of cancer cell-specific Ca^2+^ channel expression and changes, is necessary to make Ca^2+^ signaling a therapeutic target for cancer.

### Supplementary Information

Supplementary Information 1.

Supplementary Information 2.

## Data Availability

All data are available in the main text or the supplementary materials.
