# Supplementary material for: Calcineurin-mediated dephosphorylation enhances the stability and transactivation of c-Myc
Source: Sci Rep. 2023 Aug 12;13:13116. doi: 10.1038/s41598-023-40412-1 (PMC10423207; doi:10.1038/s41598-023-40412-1)
Supplement: Supplementary file 1 — Supplementary Information 1. [file 41598_2023_40412_MOESM1_ESM.pdf]

Supporting information

Table S1

Target sequences for lentivirus-based shRNA.

Gene	Sequence (5’-3’)
Luciferase	CGTGCGTGGAATGCTTCGA
FBXW7-1	CCAGAGACTGAAACCTGTCTA
FBXW7-2	CCAGAGAAATTGCTTGCTTTA
PPP3CA-1	GCGTATATGATGCCTGTATGG
PPP3CA-2	GCCAAGGGCTTAGACCGAATT

Table S2

Antibodies used in this study.

Antigen	Catalog No.	Company
β-actin	ab6276	Abcam
Calcineurin Aα	ab52761	Abcam
c-Myc	cs5605	Cell Signaling Technology
c-Myc	ab32072	Abcam
c-Myc-pThr^{58}	ab185655	Abcam
c-Myc-pSer^{62}	ab185656	Abcam
Fbxw7	A301-720A	Thermo Fisher Scientific
FLAG	M185-3	MBL
HA	PAB10343	Abnova
Hsp90	Sc13119	Santa Cruz Biotechnology

Table S3

Primer sequences for RT-qPCR.

Gene	Forward (5’-3’)	Reverse (5’-3’)
18S rRNA	GTAACCCGTTGAACCCCATT	CCATCCAATCGGTAGTAGCG
DUSP2	TGTGGAGGACAACCAGATGGTG	GAGGTATGCCAGACAGATGGT
LSM2	CTGACATCAGTGTCACAGACCC	CGCATCCTGTAGCAACTGTGTG
MRPL9	ACCAGAAGAGCCTATCACACGG	GGGCTTCTCAAAGTTCACGACAG
MYC	TTCGGGTAGTGGAAAACCAG	CAGCAGCTCGAATTTCTTCC

Table S4

Functional prediction and annotation of PPP3CA P484S/L mutations by dbNSFP v4.

Protein change	SIFT4G		Polyphen2 HDIV		Polyphen2 HVAR		MutationTaster		MutationAssessor		FATHMM-XF		MutPred Top5 features
	Score	Prediction	Score	Prediction	Score	Prediction	Score	Prediction	Score	Prediction	Score	Prediction	
p.P484S	0.001	Deleterious	1	Probably damaging	1	Probably damaging	1	Disease-causing	4.03	High	0.906	Deleterious	Gain of phosphorylation at P484 (P = 0.0023); Loss of catalytic residue at P484 (P = 0.0027); Loss of glycosylation at P484 (P = 0.0832); Gain of MoRF binding (P = 0.0938); Loss of helix (P = 0.1299)
p.P484L	0.001	Deleterious	1	Probably damaging	1	Probably damaging	1	Disease-causing	4.03	High	0.897	Deleterious	Loss of glycosylation at P484 (P = 0.064); Loss of helix (P = 0.1299); Gain of MoRF binding (P = 0.134); Loss of disorder (P = 0.2028); Gain of loop (P = 0.2045)

Somatic mutation data were obtained from COSMIC. Functional prediction and annotation of missense mutations were performed using dbNSFP v4.
