# Supplementary material for: Calcineurin-mediated dephosphorylation enhances the stability and transactivation of c-Myc
Source: Sci Rep. 2023 Aug 12;13:13116. doi: 10.1038/s41598-023-40412-1 (PMC10423207; doi:10.1038/s41598-023-40412-1)
Supplement: Supplementary file 2 — Supplementary Information 2. [file 41598_2023_40412_MOESM2_ESM.pdf]

Supplementary information

Calcineurin-mediated dephosphorylation enhances the stability and transactivation of c-Myc

Takahiro Masaki, Makoto Habara, Shunsuke Hanaki, Yuki Sato, Haruki Tomiyasu, Yosei Miki, and Midori Shimada

Figure 1

c

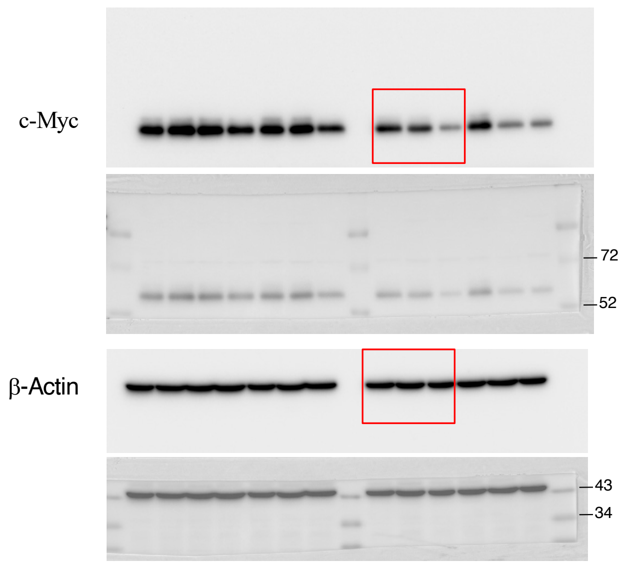

f

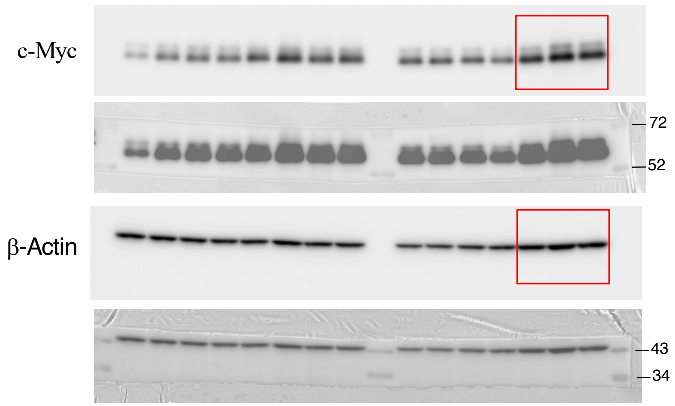

Supplemental Figure S1

Full unedited images for Figure 1 and Figure 3 are shown.

Figure 3

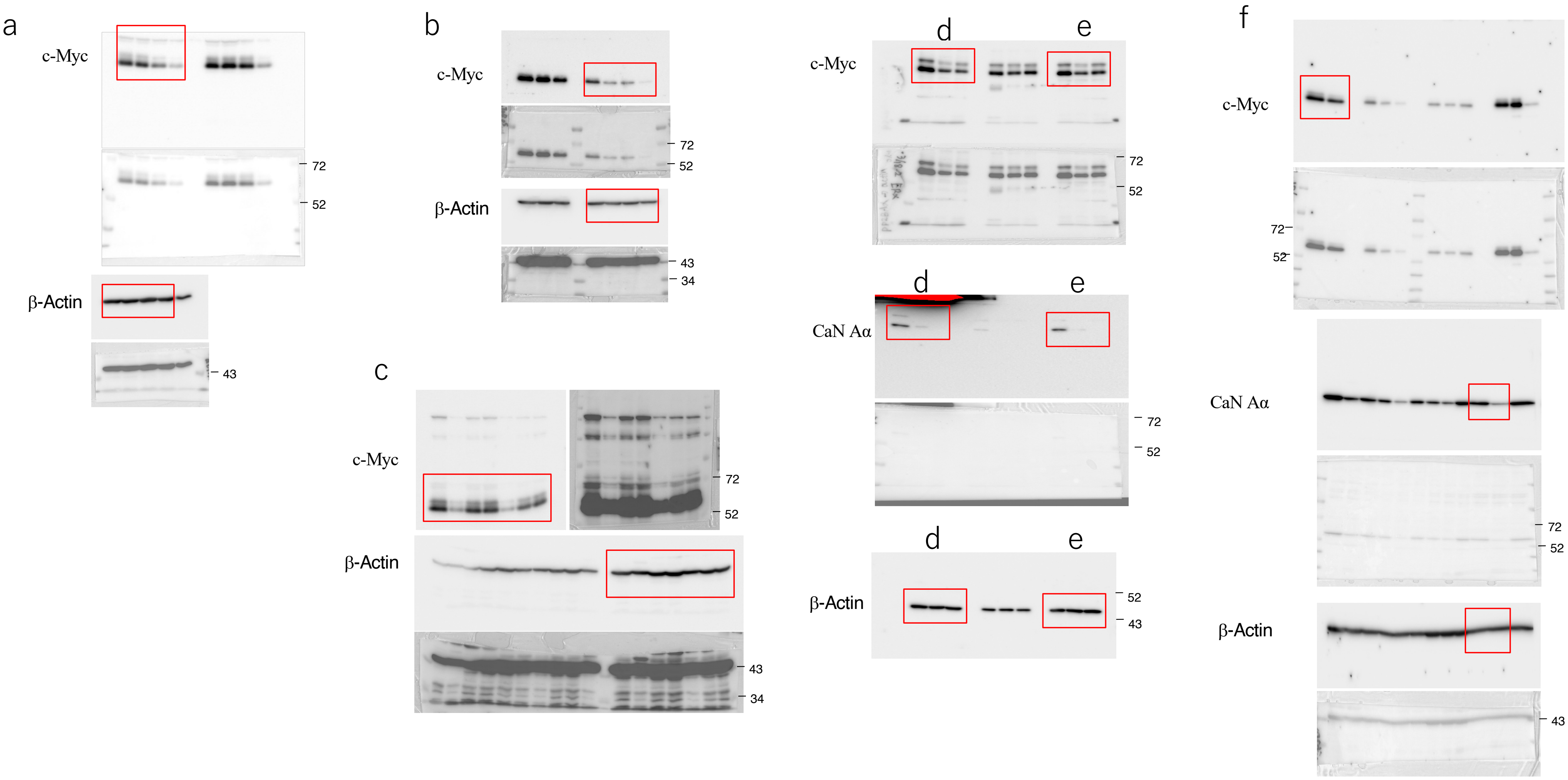

Supplemental Figure S2

Full unedited images for Figure 3 are shown.

Figure 4

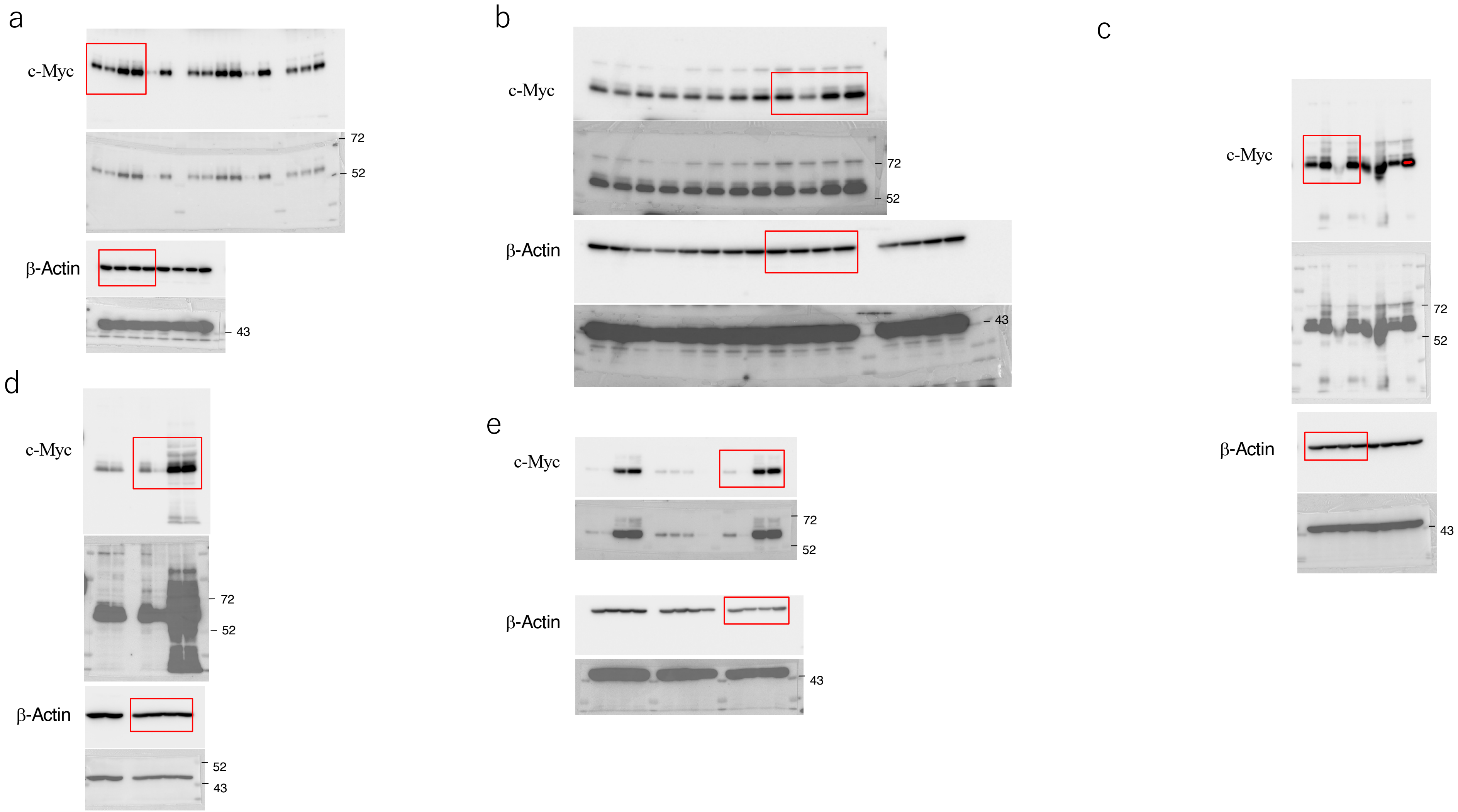

Supplemental Figure S3
Full unedited images for Figure 4 are shown.

Figure 5

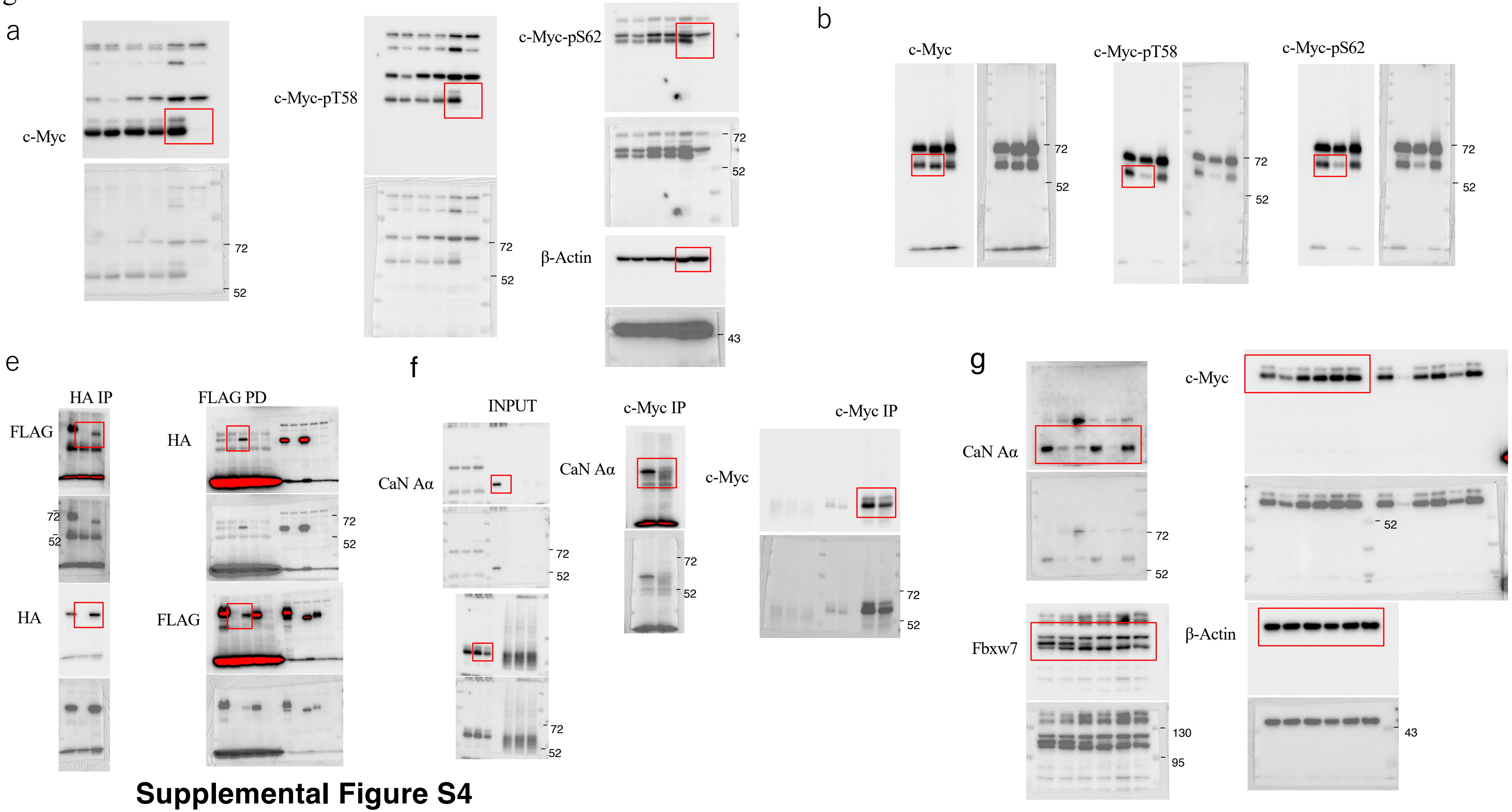

Supplemental Figure S4
Full unedited images for Figure 5 are shown.

Figure 6

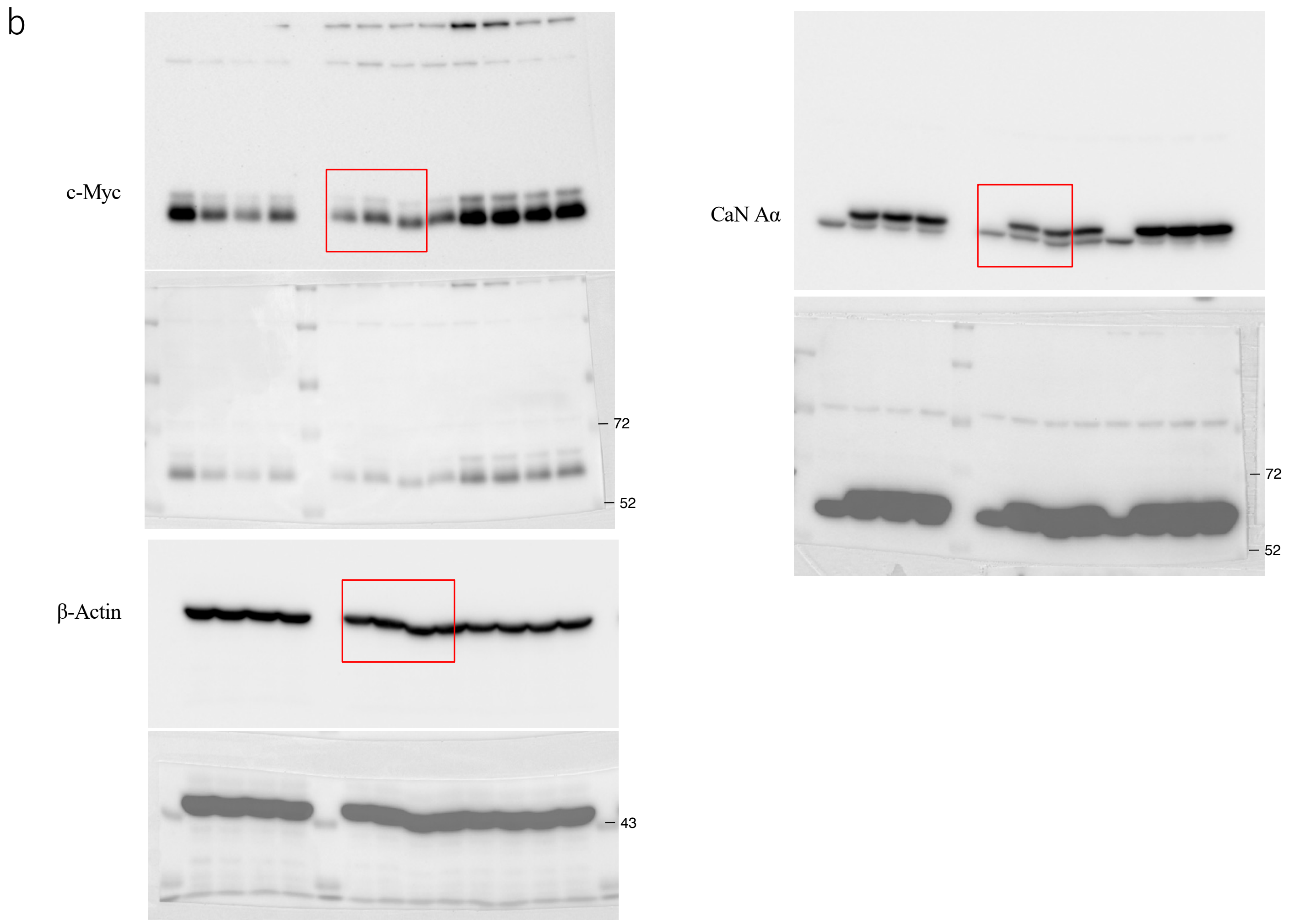

Supplemental Figure S5
Full unedited images for Figure 6 are shown.

Figure 7

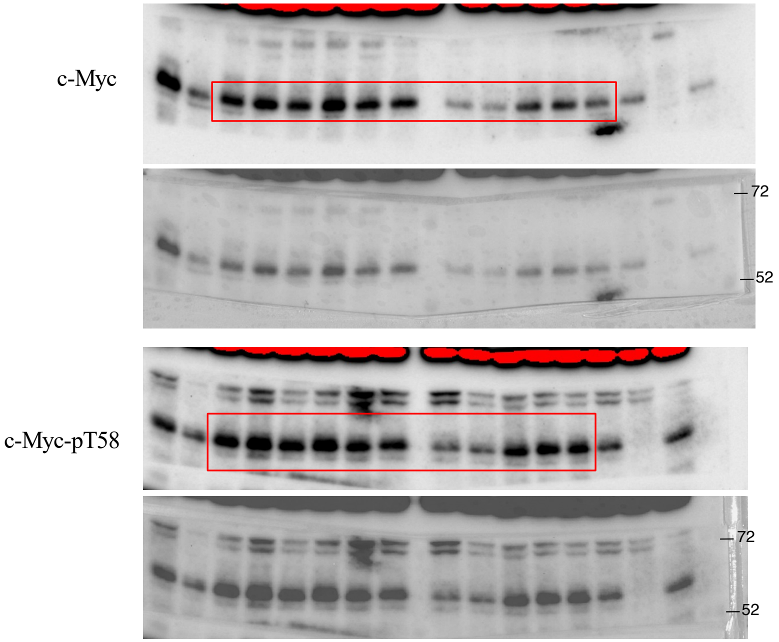

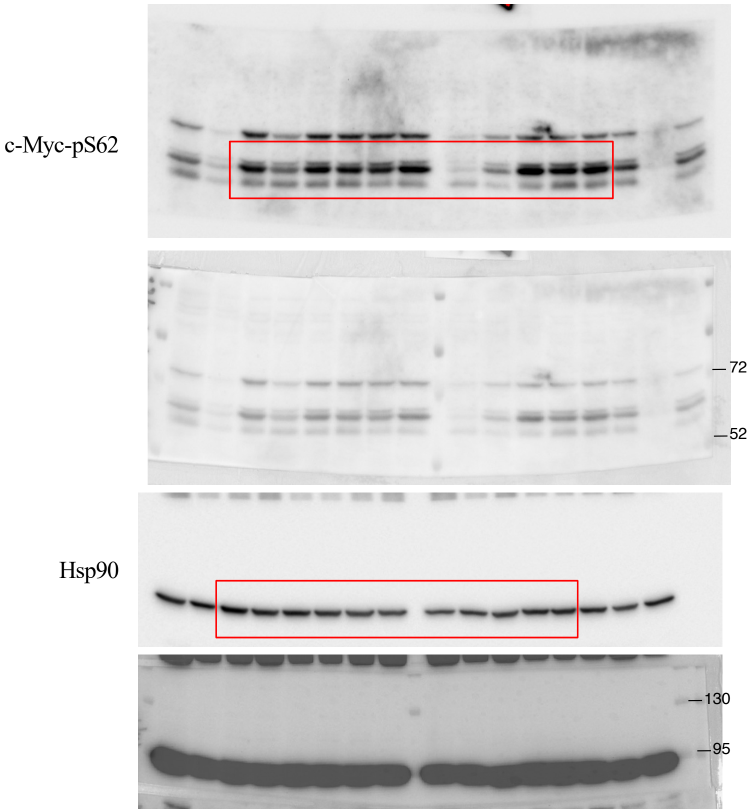

Supplemental Figure S6
Full unedited images for Figure 7 are shown.
